# Origins of Baseline Drift and Distortion in Fourier Transform Spectra

**DOI:** 10.3390/molecules27134287

**Published:** 2022-07-03

**Authors:** Feng Zhang, Xiaojun Tang, Lin Li

**Affiliations:** 1 Electronic Information Engineering, Xi’an Technological University, Xi’an 710021, China; zhang_feng@xatu.edu.cn (F.Z.); linli0624@xatu.edu.cn (L.L.); 2 State Key Laboratory of Electrical Insulation & Power Equipment, Xi’an Jiaotong University, Xi’an 710049, China

**Keywords:** baseline distortion, Fourier transform spectra, baseline correction

## Abstract

The spectrum scanned by a Fourier transform spectrometer (FTIR) often has a baseline drift. However, baseline distortion rarely occurs in a laboratory owing to the insignificant effects of environmental vibrations and electromagnetic factors. Even if it occurs, the distorted spectrum can be manually eliminated. However, in a complex environment, especially after the long-term operation of a spectrometer, the scanned spectrum may be distorted to different degrees. Herein, the origins of spectral baseline drifts and distortions are analyzed and simulated using MATLAB; furthermore, a baseline correction method based on the baseline-type model is proposed. The results of experiments performed on the methane spectrum confirm that the proposed method outperformed the improved modified multi-polynomial fitting and iterative averaging methods.

## 1. Introduction

Fourier transform infrared spectroscopy has widespread applications because it offers the advantages of fast analysis, no carrier gas, and no need for gas separation or maintenance; moreover, it can detect a variety of gases and is applicable in flammable and explosive environments [1,2,3,4]. When the spectrometer is used to obtain the sample spectrum, the background spectrum is scanned first, followed by the scanning of the sample spectrum. The background spectrum is simply the wavelength distribution of the light source of the spectroscopy. The sample spectrum should be the same as the background spectrum in the region without absorption and lower than the background spectrum in the region with absorption. The transmittance spectrum is the ratio of the sample spectrum to the background spectrum, and the absorbance spectrum is obtained by taking the negative common logarithm. 

If the optical system of the spectrometer is consistent during background and sample spectral scanning, the transmittance spectrum baseline obtained in the no-absorption region should be 1, and the logarithmically converted absorbance spectrum baseline should be 0. However, changes in the optical system during background and sample spectral scanning can cause a baseline drift and an abnormal distortion. For example, during scanning, the temperature of the light source changes, and the moving mirror tilts, which can cause a baseline drift and even lead to a baseline distortion. The absorbance value is a key factor in the field of quantitative and qualitative analysis. When a baseline drift or distortion occurs, the absorbance changes to varying degrees, thus leading to inaccurate or even incorrect quantitative analysis; therefore, it is necessary to correct the baseline drift. Several baseline drift-correction methods, such as the wavelet transform method [5,6,7,8], polynomial fitting [9,10,11,12], and penalized least squares [13,14,15,16,17], have been proposed in recent years. However, one or more parameters typically require optimization in these methods. For example, the optimum wavelet basis, decomposition lever, and wavelet coefficient threshold need to be selected in the wavelet transform method; the order of the polynomial and the smoothing parameters need to be optimized in the polynomial and penalized least square methods, respectively. Therefore, the application of these baseline correction methods in the field of online analysis is difficult.

Although Salomaa et al. have studied the origin of baseline errors [18], they have only analyzed the causes of baseline drifts for a constant light source temperature change and moving mirror tilting angle. In this study, the origins of the baseline drift and distortion in Fourier transform spectra are analyzed and simulated using MATLAB. Next, according to the mathematical model of the baseline drift, a baseline correction method to correct the drift spectrum was proposed. Furthermore, the performance of the proposed method is tested on the methane spectrum. We have proposed an approach to address the problem of spectral baseline distortion in our previous work [19].

## 2. Origins of Baseline Drift and Distortion

After the long-term operation of a spectrometer, the performance of its optical components declines. In this study, we analyzed and simulated the origins of the baseline drift and distortion caused by changes in the light source temperature, moving mirror tilt, interferometer modulation, laser wavelength, and a loss of interference signals.

### 2.1. Light Source Temperature Change

A carbon-silicon rod, ceramic, and a metal wire are used as a mid-infrared light source in an infrared spectrometer. The energy radiated from these materials determines the detection sensitivity of the spectrometer. One part of the light source energy is converted into heat energy and the other part into radiant energy. 

According to Planck’s radiation theory, the radiation intensity of a blackbody is related to its temperature and radiation efficiency. The radiation efficiency of a spectrometer is a constant. Therefore, the temperature of the light source determines its radiation intensity. Assuming that the light source temperature is *T*_0_ when the background spectrum is scanned, the wavenumber distribution of the radiated power density of the light source can be expressed as

(1) $$W(v)=\frac{2hc^{2}v^{3}}{\exp(hcv/kT_{0})-1}$$

where *v* is the wavenumber, *h* is the Planck’s constant, *c* is the velocity of light, and *k* is the Boltzmann constant. Suppose that the light source temperature changes to *T*_1_ when the spectrum of the sample is scanned; then, the wavenumber distribution is given by

(2) $$W(v)=\frac{2hc^{2}v^{3}}{\exp(hcv/kT_{1})-1}$$

Assuming that the total efficiency of the spectrometer is *ε*, the transmittance spectrum can be calculated as

(3) $$T(v)=\frac{\int_{-\infty }^{\infty }\int_{-\infty }^{\infty }\frac{2\varepsilon hc^{2}v^{3}}{\exp(hcv/kT_{1})-1}\cos(2\pi v\delta )dvd\delta }{\int_{-\infty }^{\infty }\int_{-\infty }^{\infty }\frac{2\varepsilon hc^{2}v^{3}}{\exp(hcv/kT_{0})-1}\cos(2\pi v\delta )dvd\delta }$$

where *δ* is the optical path difference. If the background and sample spectra are scanned at temperatures of *T*_0_ and *T*_1_, respectively, and remain unchanged, then the transmission spectrum can be calculated as

(4) $$T(v)=\frac{\frac{2\varepsilon hc^{2}v^{3}}{\exp(hcv/kT_{1})-1}}{\frac{2\varepsilon hc^{2}v^{3}}{\exp(hcv/kT_{0})-1}}\approx \frac{\exp(\frac{hcv}{kT_{0}})}{\exp(\frac{hcv}{kT_{1}})}$$

In Equation (4), *hcv*/*kT* is assumed to be significantly greater than one. To obtain the absorbance spectrum, we take a negative common logarithm of Equation (4). Thus, Equation (4) can be expressed as

(5) $$A\approx \mathrm{lg}\frac{\exp(hcv(T_{0}-T_{1}))}{kT_{0}T_{1}}=0.4343\frac{hcv(T_{0}-T_{1})}{kT_{0}T_{1}}$$

Equation (5) indicates that when the temperature of the scanned background is different from that of the sample and remains constant during scanning, the absorbance spectrum baseline and wavenumber are approximately linear. If *T*_1_ > *T*_0_, the baseline is inclined downward; otherwise, it is inclined upward.

If the background spectrum was scanned at a temperature of *T*_0_ and remained unchanged while the light source temperature is impacted by the voltage during sample spectral scanning, the temperature would rise or fall temporarily. If the light source temperature changes in this short period of time, the corresponding spectrum baseline will be distorted to different degrees. The transmittance spectrum can be calculated as

(6) $$T(v)=\frac{\int_{-\infty }^{\infty }\int_{-\infty }^{\infty }\frac{2\varepsilon hc^{2}v^{3}}{\exp(hcv/kT_{1})-1}\cos(2\pi v\delta )dvd\delta }{\frac{2hc^{2}v^{3}}{\exp(hcv/kT_{0})-1}}$$

where **T**_1_ (a vector) is the light source temperature when sampling the interference data. If the number of sampled interference signals is n, the length of **T**_1_ is n.

**Figure 1:**
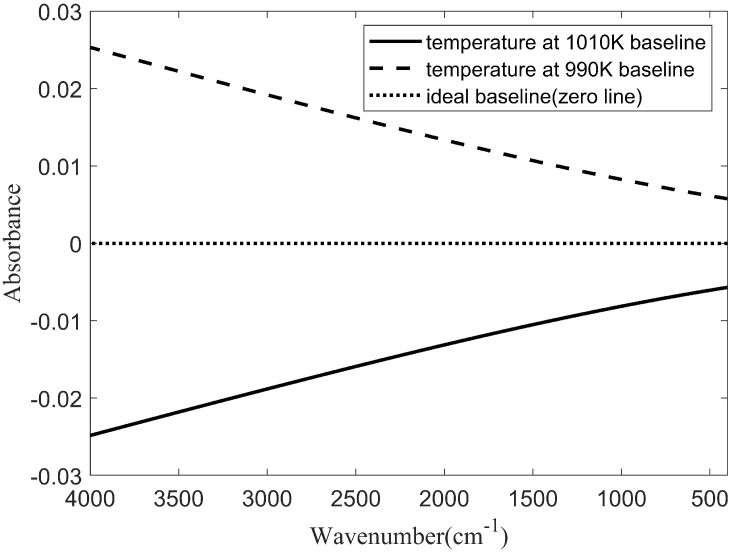
Ideal baseline and baseline at 990 K and 1000 K.

In this study, we simulated the influence of changes in the light source temperature on the baseline. All the simulation results were obtained in MATLAB 9.1.0 (R2016b). In the simulation, the light source temperature was set at 1000 K and remained unchanged during background scanning. When the sample spectrum was scanned, the changes in light source temperature were as follows: an increase of 10 K and a decline of 10 K. In the simulation, the sampling interval of the interference signal was 850 nm; the interference signals were sampled at both ends, and a total of 8191 interference signals were sampled. The ideal baselines and the baselines at 990 K and 1000 K are shown in Figure 1.

As seen in Figure 1, when the temperature of the light source was increased by 10 K, the baseline was lower than the ideal baseline, and the deviation from the ideal baseline in the high wavenumber region was greater than that in the low-wavenumber region. When the temperature of the light source decreased, the baseline was higher than the ideal baseline. Similarly, the deviation of the high wavenumber region was greater than that of the low-wavenumber region. The baseline shape is approximately a straight line, and the linearity is 4.52%. After careful observation, it can be found that the baseline linearity in the low wavenumber is higher than in the high wavenumber. This is because, with the decrease in the wavenumber, the value of the exponential function (exp (*hcv*/*kT*)) becomes smaller, resulting in the baseline drift not strictly in accordance with the linear relationship. 

**Figure 2:**
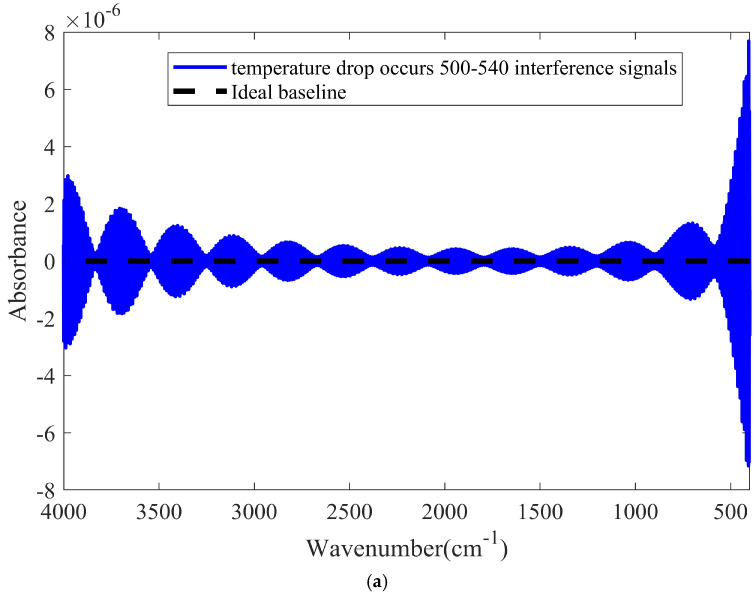

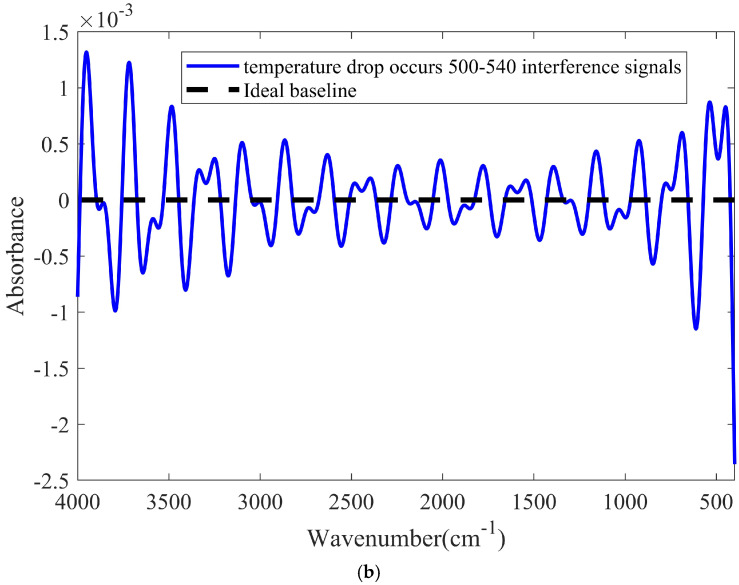
Simulated baselines with the light source temperature decreasing for a short time. (**a**) The temperature drop of the light source occurs far from zero optical path difference. (**b**) The temperature drop of the light source occurs near the zero optical path difference.

During spectral scanning, the voltage shock led to a temporary change in the light source temperature. In the simulation, the light source temperature was affected at 50 ms and then reduced to 800 K. We considered 10 s as the period of the spectrometer scanning of 8191 interference signals. Currently, commercial spectrometers typically take approximately 10 s to complete one scan. Therefore, 41 interference signals were affected within 50 ms. The temperature of the light source decreased for a short time change was simulated. One is the temperature of light source decrease occurring in a region far from the zero optical path difference; the other is the temperature decrease occurring near the zero optical path difference. The simulated baselines are shown in Figure 2.

As shown in Figure 2, if the temperature drop occurs near the zero optical path difference, the baseline will fluctuate significantly. Moreover, the fluctuation amplitudes in the high and low-wavenumber regions are larger than that in the medium-wavenumber region owing to the lower radiation power density of the light source at the high and low-wavenumber regions. It can also be seen that when the baseline is distorted, its shape is approximately sinusoidal.

### 2.2. Moving Mirror Tilt

**Figure 3:**
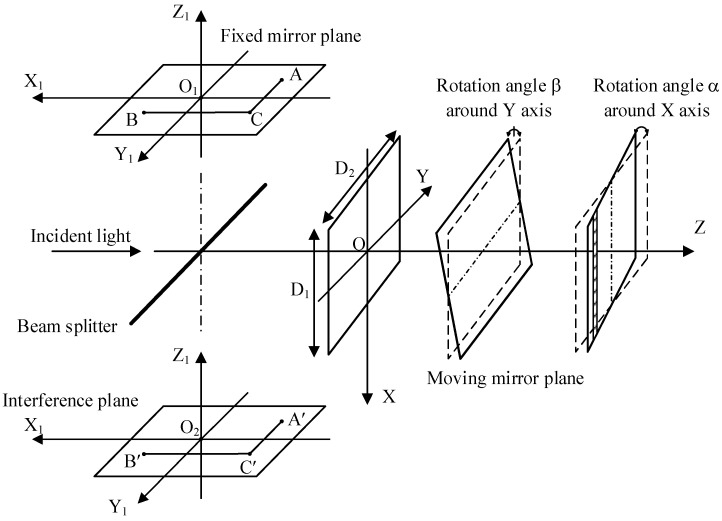
Structures of a fixed mirror and moving mirror in the interferometer.

During spectral scanning, the moving mirror inevitably tilts, which causes the parallel error between the moving and fixed mirrors, leading to the change in the interferometer modulation. According to the shape of the beam aperture, the beam incident on the mirror is rectangular or circular. In this study, we analyzed the influence of the moving mirror tilt with a circular beam on the baseline. The structures of the fixed and moving mirrors in the interferometer are shown in Figure 3.

When the tilt angles *α* and *β* are nonzero at the same time, the optical path of the light source arriving at the interference plane changes, leading to the change in modulation. The normal vector of the moving mirror plane is

(7) i=(−cosαsinβ,sinαcosβ,−cosαcosβ)

The center coordinates on the moving mirror plane are considered to be (0,0,0) when the moving mirror is tilted along the X and Y axes. In this case, according to the plane equation, the following equation can be obtained

(8) −xcosαsinβ+ysinαsinβ−zcosαcosβ=0

where x ∈ [−*D*_1_/2, *D*_1_/2] and y ∈ [−*D*_2_/2, *D*_2_/2]. When the mirror is tilted, *α* and *β* are very small; thus, cos*α* ≈ 1, sin*α* ≈ *α*, cos*α* ≈ *β*, and sin*β* ≈ *β*. Equation (8) can be simplified as

(9) −βx+αy−z=0

The optical difference is given by

(10) Δσ=2Δz=2αy−2βx

The interference signals from the whole interference plane can be expressed as

(11) $$\begin{array}{ll} I(z) & =\frac{1}{D_{1}D_{2}}\int_{-D_{1}/2}^{D_{1}/2}\int_{-D_{2}/2}^{D_{2}/2}I\left(\sigma +2\alpha y-2\beta x\right)dxdy\\ & =\frac{1}{D_{1}D_{2}}\int_{-D_{1}/2}^{D_{1}/2}\int_{-D_{2}/2}^{D_{2}/2}\int_{-\infty }^{\infty }E(v)\exp\left(i2\pi v\left(\sigma +2\alpha y-2\beta x\right)\right)dvdxdy\\ & =\frac{1}{D_{1}D_{2}}\int_{-\infty }^{\infty }E(v)\exp(i2\pi v\sigma )\int_{-D_{1}/2}^{D_{1}/2}\exp\left(-i2\pi v\times 2\beta x\right)dx\int_{-D_{2}/2}^{D_{2}/2}\exp\left(i2\pi v\times 2\beta y\right)dydv\\ & =\int_{-\infty }^{\infty }E(v)\exp(i2\pi v\sigma )\sin c\left(2\pi v\beta D_{1}\right)\sin c\left(2\pi v\alpha D_{2}\right)dv \end{array}$$

where sinc(*x*) = sin(*x*)/*x*. If the moving mirror does not tilt when scanning the background spectrum, the background spectrum is *E*(*v*), and if the moving mirror tilts at a constant angle during sample spectrum scanning, the absorbance spectrum is calculated as

(12) $$A(v)=-\mathrm{lg}\left[\frac{E(v)\mathrm{sinc}\left(2\pi v\beta D_{1}\right)\mathrm{sinc}\left(2\pi v\alpha D_{2}\right)}{E(v)}\right]=-\log10\left[\mathrm{sinc}\left(2\pi v\beta D_{1}\right)\mathrm{sinc}\left(2\pi v\alpha D_{2}\right)\right]$$

We can approximate it by the Taylor series expansion as

(13) $$A\approx -\mathrm{lg}\left\{\left[1-\frac{{\left(2\pi v\beta D_{1}\right)}^{2}}{3!}+\frac{{\left(2\pi v\beta D_{1}\right)}^{4}}{5!}+\cdots \right]•\left[1-\frac{{\left(2\pi v\alpha D_{2}\right)}^{2}}{3!}+\frac{{\left(2\pi v\alpha D_{2}\right)}^{4}}{5!}+\cdots \right]\right\}$$

Using the series expansion of ln(1 + *x*) and removing the terms with orders higher than four, the absorbance spectrum can be calculated as

(14) $$A\approx 0.4343\left[\frac{{\left(2\pi v\beta D_{1}\right)}^{2}}{6}+\frac{{\left(2\pi v\beta D_{1}\right)}^{4}}{180}+\frac{{\left(2\pi v\alpha D_{2}\right)}^{2}}{6}+\frac{{\left(2\pi v\alpha D_{2}\right)}^{4}}{180}\right]$$

Equation (13) indicates that the absorbance spectrum baseline is a polynomial combination of the even-degree terms of the wavenumber, and its baseline is parabolic. It shows that if the moving mirror is tilted at a fixed angle when scanning the sample spectrum, the baseline will drift upward.

**Figure 4:**
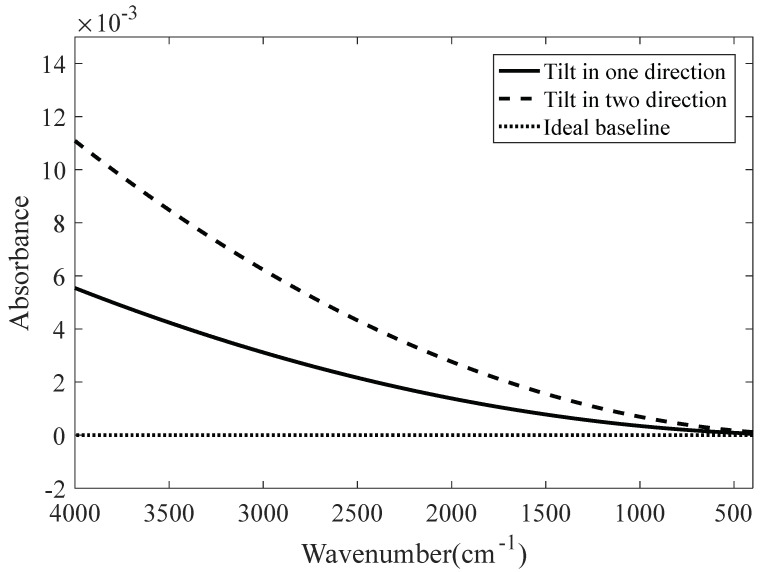
Moving mirror at a fixed tilt angle.

**Figure 5:**
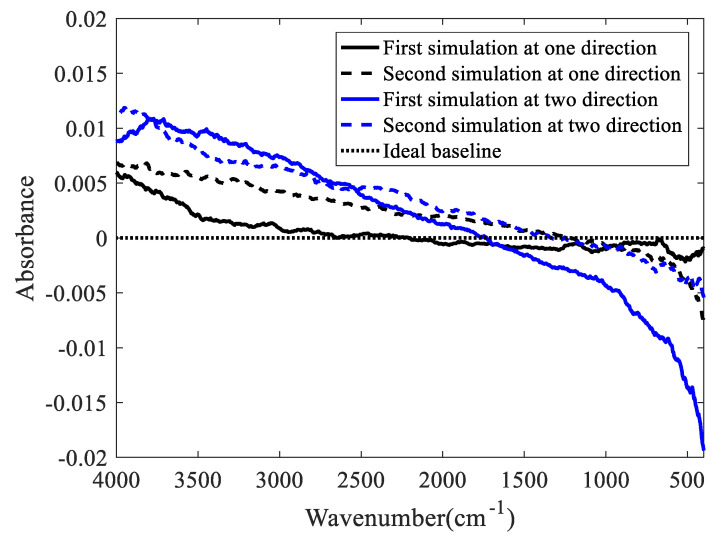
Moving mirror at a random tilt angle.

The influence of the moving mirror tilt on the baseline is simulated using MATLAB. In the simulation, it is considered that the moving mirror does not tilt when scanning the background spectrum. When the sample spectrum is scanned, the moving mirror is tilted as follows: 0.24″ in one direction; 0.24″ in two directions; randomly in one direction, with a tilt angle range of 0~0.24″; and randomly in two directions, with a tilt angle range of 0~0.24″. Figure 4 and Figure 5 show the relationship between the wavenumber and absorbance when the tilt angles are fixed and random, respectively. 

As shown in Figure 4 and Figure 5, the deviation from the ideal baseline when the moving mirror tilts in two directions is approximately twice as much as that when the moving mirror tilts in one direction. Similarly, the deviations from the ideal baseline in the high- and low-wavenumber regions are larger than that in the medium-wavenumber region.

### 2.3. Interferometer Modulation Change

**Figure 6:**
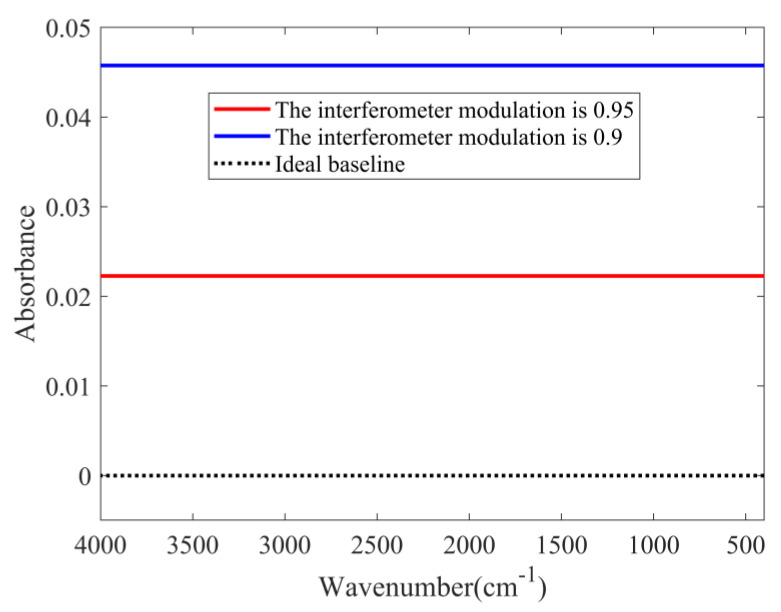
Baselines were obtained when the interferometer modulation is reduced to 0.95 and 0.9.

The Fourier transform spectrometer (FTIR) can also be affected by mechanical vibration (external impact, dust in the optical path), which can lead to a change in the interferometer modulation. If the modulation changes during the entire scanning process, the baseline can shift upward. If the modulation changes temporarily, the baseline can be distorted. In this simulation, the light source temperature is 1000 K. A total of 8192 interference signals are sampled, and 41 interference signals are affected when the modulation changes temporarily. During the spectrum scanning, the baselines obtained when the interferometer modulation is reduced to 0.95 and 0.9 are shown in Figure 6.

**Figure 7:**
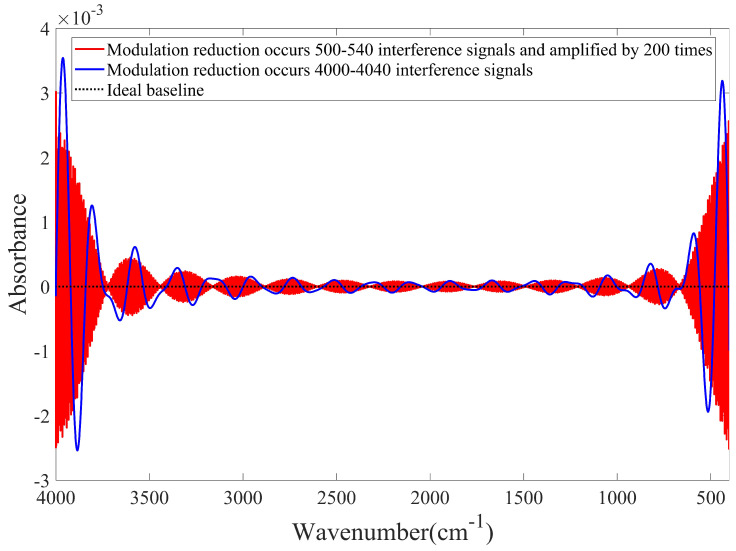
Baselines were obtained when the interferometer modulation is reduced to 0.95 and 0.9.

Figure 6 indicates that if the interferometer modulation decreases during the entire scanning process, the baseline will shift upward, and the shift amplitude depends on the modulation degree. The simulated baselines for the temporary modulation changes are shown in Figure 7.

Figure 7 clearly indicates that when the modulation changes temporarily, the baseline is distorted to different degrees. If the modulation change occurs near the region of zero optical path difference, the baseline fluctuates significantly. In addition, the fluctuation amplitudes in the high- and low-wavenumber regions are larger than those in the medium-wavenumber region because the interference signal is the strongest near the region of zero optical path difference; the interference signal near the region of zero optical path difference is significantly influenced when the interferometer modulation changes. Similarly, when the baseline is distorted, it is approximately sinusoidal.

### 2.4. Laser Wavelength Change

**Figure 8:**
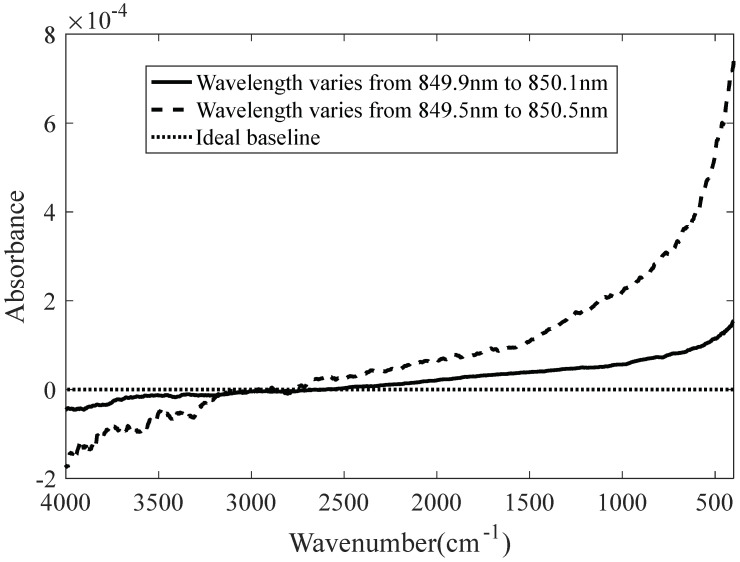
The baseline varies with respect to the laser wavelength.

The interference signals are sampled according to the laser wavelength; in the simulation, the laser wavelength varies from 849.9 to 850.1 nm and 849.5 to 850.5 nm. The simulation results are presented in Figure 8.

### 2.5. Interference Signal Missing Sampling

**Figure 9:**
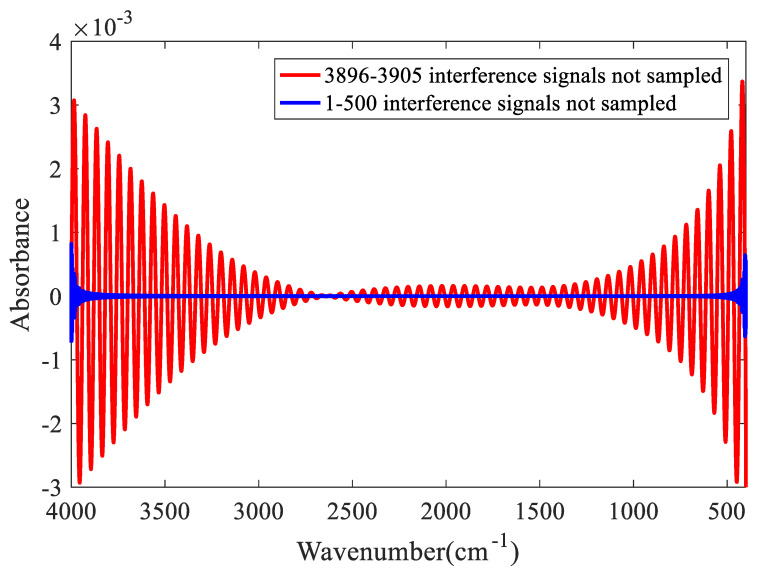
Influence of missing interference signal sampling on the baseline.

When the spectrometer is used, the voltage fluctuation causes the trigger sampling signal to be lower than the threshold voltage of ADC sampling, or there are dust particles in the moving mirror rail, which can lead to missing interference signals. The simulation results are presented in Figure 9.

Figure 9 shows that if the missing interference signals occur near the region of zero path difference, the baseline will fluctuate significantly. It is noteworthy that during spectrum scanning, the phenomenon of missing interference signals can easily occur in regions far away from that of zero path difference because the interference signals are very small and get mixed together with the noise signals in these regions. Although the resolution of ADC is low enough for most commercial FTIR, the noise cannot be identified and separated from the signals.

## 3. Baseline Drift-Correction Method

Through this analysis, when the baseline drifts, the baseline type can be approximately expressed as 0, 1, 2, and 4 times of the wavenumber. Therefore, the baseline-type model can be represented as follows

(15) $$b(v)=c_{4}v^{{}_{4}}+c_{2}v^{{}_{2}}+c_{1}v+c_{0}$$

Herein, we propose an automatic baseline correction method based on the baseline-type model (BTM). The core of the BTM is that the polynomial in the improved modified multi-polynomial fitting (I-ModPoly) [20] method is represented by Equation (15), and the DEV calculation in the I-ModPoly method is changed to

(16) DEV=std((O(v)−P(v))<0)

where *O*(*v*) is the original spectrum, *P*(*v*) is the polynomial fitting baseline, *v* is the wavenumber, and std is the standard deviation function. Thus, the problem of a boosted fitted baseline in the I-ModPoly method due to the overestimation of the noise signal can be avoided, and the highest order of polynomials need not be optimized in the I-ModPoly method.

## 4. Experiments

**Figure 10:**
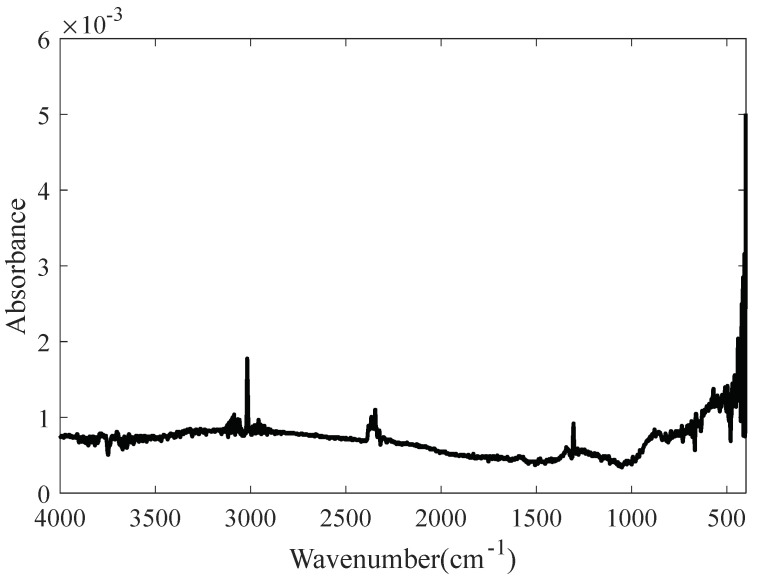
Spectrum of methane with a concentration of 0.002% before baseline correction.

Methane is an important characteristic gas in coal mines. In this study, the methane spectrum was scanned by a Fourier transform infrared spectrometer; the type of spectrometer used was Spectrum Two, produced by Perkin Elmer, Waltham, United States. The optical path was 10 cm, the spectral resolution was set at 1 cm^−1^, and the scanning range was 400~4000 cm^−1^. The spectrum of methane with a concentration of 0.002% is shown in Figure 10.

**Figure 11:**
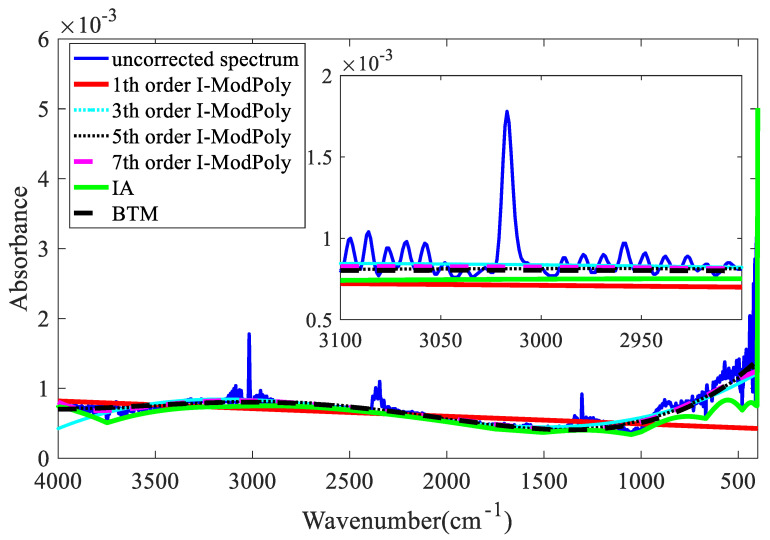
Baselines corrected by BTM and the IA and I-ModPoly methods.

Figure 10 shows that the scanned spectrum has a baseline shift. This was corrected by BTM and the iterative averaging (IA) [21] and I-ModPoly methods, and the corrected baselines for the different highest orders are shown in Figure 11.

As shown in Figure 11, the IA method tends to pass through the lowest point of the spectrum, resulting in different degrees of underfitting in the entire spectrum; the baselines obtained by the I-ModPoly method with highest orders of one and three have clear errors. In the main absorption peak region for methane, the baseline fitted by I-ModPoly is higher than the real baseline. The baseline obtained by the I-ModPoly method is higher than that obtained by the BTM owing to the overestimation of noise in the I-ModPoly method. In addition, the BTM is based on the BTM to fit the baseline, and the highest order needs to be optimized in the I-ModPoly method. We can thus confirm that the BTM is better than the I-ModPoly method for baseline correction. 

## 5. Conclusions

In this study, the origins of the spectral baseline drift and distortion were analyzed and simulated using MATLAB. The results show that when the light source temperature and moving mirror tilt angle changed constantly, the spectral baseline drifted, whereas when they changed randomly, the spectral baseline was distorted. The change in the reflectance of the beam splitter caused the baseline to shift upward or downward. The change in the laser wavelength and the missing interference signal also caused the baseline distortion. To address the problem of spectral baseline drift, a baseline correction method based on the BTM was also proposed. The results of experiments performed on the methane spectrum confirm that the proposed method outperformed the improved modified multi-polynomial fitting and IA methods. An identification and treatment approach based on the shape and distribution of the baseline has been reported in our previous work to address the problem of spectral baseline distortion [19]. 

## Data Availability

The data in the paper can be obtained from the authors.
